# Identifying Factors That Might Affect Outcomes of Exercise-Based Therapies in Long-COVID

**DOI:** 10.3390/diseases12110293

**Published:** 2024-11-15

**Authors:** Anna-Lena Krüger, Björn Haiduk, Marijke Grau

**Affiliations:** 1 Institute of Cardiovascular Research and Sports Medicine, Molecular and Cellular Sports Medicine, German Sport University Cologne, 50933 Cologne, Germany; 2 S.P.O.R.T. Institut, Institute of Applied Sports Sciences, Lindlarer Strasse 95, 51491 Overath, Germany

**Keywords:** Long-COVID, rehabilitation, exercise, physical activity, physical fitness, fatigue, pulmonary impairments, quality of life

## Abstract

Background: Long-COVID, which might develop after a SARS-CoV-2 infection, is a rather new disease without standardized treatment strategies. A large number of approaches that integrate physical activity have been described in the literature, and this systematic review aims to examine changes in symptom severity, physical fitness, respiratory symptoms and quality of life during training and identify factors that might influence the respective outcomes. Methods: A literature search was conducted using the databases Pubmed, PEDro, BioMed Central, EBSCOhost, ProQuest and the ZBSport from 13 February 2024 to 27 February 2024, and 39 studies fulfilled the search criteria. Results: The analyzed study designs varied regarding the type of intervention (isolated vs. multidisciplinary), duration and intensity of training sessions and overall length of the program. Individualized holistic concepts of physical activity paralleled by additional approaches demonstrated high effectiveness. However, many of the participants continue to suffer from Long-COVID after the intervention. Conclusions: Long-COVID treatment should be individualized, multifactorial and not limited in time and should consider each patient’s pre-existing conditions and individual course of the disease to provide the best possible support and care.

## 1. Introduction

COVID-19 is an infectious disease caused by the coronavirus SARS-CoV-2 (severe acute respiratory syndrome coronavirus type 2), which was first described in 2019 and then developed into the COVID-19 pandemic. So far, ~ 776 million people worldwide have been infected with SARS-CoV-2 [1]. Various long-term health effects have been associated with a previous SARS-CoV-2 infection. These are summarized under the term “Long-COVID” and describe health complaints that persist or reappear beyond the acute phase (4 weeks to 12 weeks) of a SARS-CoV-2 infection [2,3]. The Post-COVID-19 syndrome refers to health complaints that persist for more than 12 weeks after the onset of SARS-CoV-2 infection, which cannot be explained otherwise [3]. The term Long-COVID includes both the persistence of symptoms and Post-COVID-19 syndrome, and thus, the term Long-COVID will be used in the following description [3]. The various symptoms might overlap, fluctuate and change over time and can affect any body system and impair everyday life. According to previous studies, it might be assumed that Long-COVID shows no uniform clinical picture but rather consists of a series of long-term health consequences associated with a previous SARS-CoV-2 infection. Very common and commonly reported symptoms that can occur separately or together include muscle and joint pain (63%) [4], dyspnea at rest and during exertion (61%) [5], fatigue (58%) (tiredness, exhaustion, increased recovery time, limited resilience) [5,6,7], reduced performance/activity (54%) [8], headaches (44%) [7], cognitive impairment (27%) [7] or anxiety and depression symptoms (23%) [4]. Long-COVID occurs in all age groups, yet the age group 36–50 years shows the highest number of Long-COVID diagnoses. However, it should be noted that this age group also shows the highest overall number of COVID-19 cases. The prevalence of Long-COVID is estimated at around 3–15% after 4 to 12 weeks [9,10,11] and ~6.5% after 6 to 12 months after SARS-CoV-2 [10,11]. The underlying mechanisms of Long-COVID have not yet been completely understood, but there is evidence that chronic inflammation [12] and occlusion of small vessels (microthrombi) or microclotting [13], viral persistence [14,15], activation of the Epstein-Barr virus [16], changes in the gut microbiome [17] and autoimmune processes are involved in the development of long-term health consequences after SARS-CoV-2 infection [17].

The diagnosis of Long-COVID is based on the exclusion principle, the collection of basic criteria, such as validation of a SARS-CoV-2 infection and respective Long-COVID-associated symptoms, and a specific medical examination [18].

Because of the newness of the disease, there is still no standard treatment. However, various national guidelines [3,18,19,20,21,22,23,24,25,26,27] suggest that physical activity may be beneficial. This assumption is also based on positive experiences in the treatment of various diseases, including neurological, psychiatric, metabolic, cardiovascular or pulmonary diseases, but also cancer or musculoskeletal disorders [28,29,30]. Also, disease-related symptoms, such as fatigue, which i.a. has been reported as a side effect of cancer treatment [31], have been positively influenced by physical training [32]. Regular training is also an effective method to positively influence the quality of life, mood, stress levels, anxiety and depression, which might be altered in Long-COVID. Regular exercise can also influence the perception of pain, and finally, training is known to improve muscle strength, muscle size, functional capacity, lung capacity and systematic oxidative stress [33,34]. Therefore, the effects of exercise-based interventions have already been investigated in Long-COVID, and the respective literature suggests that physical exercise might significantly improve Long-COVID symptoms, such as fatigue and dyspnea, functional capacity and muscle strength [35,36,37,38]. Less good or no improvements are reported for other symptoms associated with Long-COVID, including anxiety, depression and cognitive performance [36,38]. However, the reports also indicate a high risk of bias in the studies reviewed [39,40]. As there are still no guidelines for standardization in the treatment of Long-COVID patients in rehabilitation, the studies to date show great heterogeneity with regard to the interventions carried out. This can be seen in the overall duration of the intervention, as well as the length of the training sessions, the type of training, the frequency and the intensity of the training sessions and their effect on Long-COVID-related symptoms.

Therefore, this review aims to elaborate on factors such as duration of exercise program, frequency and intensity of exercise sessions and initiation of treatment in relation to COVID-19 disease and highlights their impact on symptom reduction, improvement of physical fitness, respiratory symptoms and quality of life. The strengths and weaknesses of the approaches to date will be the subject of discussion.

## 2. Materials and Methods

The review was conducted in accordance with the PRISMA guidelines for systematic reviews to ensure the reproducibility of this systematic literature search [41], and the research question was derived with the help of the PICO framework.

**Figure 1:**
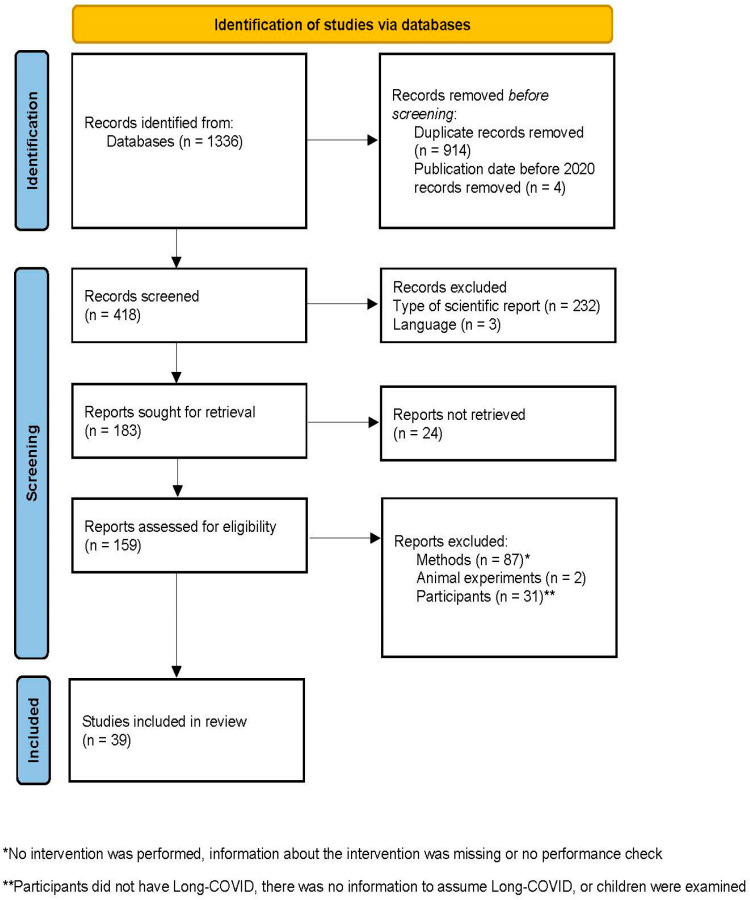
Research process flow diagram inspired by PRISMA 2020 Flow Diagram [41].

The research process is presented in a flow diagram (Figure 1). The following databases were used for the literature search: Pubmed, PEDro, BioMed Central, EBSCOhost, ProQuest and the ZBSport (Central Library of the German Sport University Cologne). The literature search was performed from 13 February 2024 to 27 February 2024. The syntax of the literature search was as follows: (Long-COVID OR Post-COVID OR long hauler OR post-acute COVID OR chronic COVID) AND (rehabilitation OR physiotherapy OR pulmonary rehabilitation OR pacing OR exercise OR physical activity OR training).

One problem identified during the literature search was the fact that not all studies used the same definition of Long-COVID. A variety of different words were used to describe the disease, such as Post-COVID, Post-COVID syndrome, post-acute sequelae of COVID-19, chronic COVID, Long-COVID or long hauler. However, even when the term Post-COVID was used, it did not always mean that patients with Post-COVID syndrome were investigated. Often, only the condition after a SARS-CoV-2 infection was meant, without any time specification. Therefore, studies were included that examined Long-COVID patients according to the following definition regardless of the terminology used: Long-COVID is defined as the continuation or development of new symptoms 4 weeks after SARS-CoV-2 infection.

The inclusion criteria for articles of these databases were as follows: (1) investigations with humans 13 years or older with symptoms of Long-COVID, (2) treatments or interventions based on physical exercise that include measurements of Long-COVID symptoms and (3) the accessibility of the full text. Exclusion criteria were as follows: (1) animal experiments, (2) children under 13 years old, (3) treatments and interventions that were carried out during the acute infection or less than 4 weeks of persistent symptoms, (4) no clear description of the intervention (5) study designs like reviews, protocols, case reports and letter to the editor, (6) home-based training like telerehabilitation without performance check, (7) publication date before 2020 and (8) papers not in English.

The keyword search in the above databases yielded *n* = 1336 results. After removing duplicates (*n* = 914) and articles that met exclusion criterion 7 (*n* = 4), *n* = 418 articles were included in the final review. After title and abstract screening, *n* = 259 articles were excluded because they further met exclusion criteria 5 and 8. The full text of the remaining *n* = 159 articles were screened by two research studies in an independent process to identify those articles that qualified for inclusion in this review according to the defined inclusion criteria. The final search yielded *n* = 39 articles. The included articles were categorized according to their treatments in physical exercise alone or combined with breathing or educational sessions, multidisciplinary rehabilitation, telerehabilitation, rehabilitation with virtual reality (VR) technology or pacing. In the studies using VR technology, participants wore VR goggles (head-mounted displays) while cycling on an ergometer or relaxing in a chair. These then played a virtual landscape with realistic elements and sound effects. The information on the study outcomes was extracted from the relevant section of the respective results section. The percent changes of the respective study parameters were determined by pre–post comparison of the reported values.

The final studies were critically assessed with regard to the following factors: (1) number of participants; (2) start of rehabilitation; (3) categories of intervention; (4) duration, frequency and intensity of training sessions; (5) effects of the intervention on Long-COVID symptoms; and (6) adverse events.

Studies were appraised for quality using CADIMA [42,43]. A rating scale from 0 to 4 was based on the following criteria: (1) sample size: ≥30 = 1; smaller sample = 0; (2) randomized controlled trials = 1; not randomized, no controls = 0; (3) studied one or more categories (symptoms, quality of life, physical fitness, pulmonary parameters) = 1; otherwise = 0; (4) standard and objective evaluation criteria = 1; otherwise = 0. Based on the criteria mentioned earlier, we rated each of the 39 studies independently in a range of 0 to 4.

## 3. Results

This systematic review included 39 studies, and the results are summarized in Table 1 (for a detailed version of this table, see Supplement Table S1). The results of the quality assessment showed that of the thirty-nine studies reviewed, 30.77% were high quality (twelve out of thirty-nine), 43.59% were moderate quality (seventeen out of thirty-nine), 23.08% were low quality (nine out of thirty-nine) and 2.56% were very low quality (one out of thirty-nine).

### 3.1. Participants

The number of participants varied widely, ranging from nine [46] to 585 [79]. Only four out of thirty-nine (4/39) studies have examined more than 100 subjects. However, three of these studies assigned the subjects to different sub-groups, thus limiting the total sample size. Twenty-eight studies investigated over 30 participants, five studies investigated less than 30 participants, and two studies investigated less than 10 subjects. These sample sizes can be considered as low, given the high heterogeneity of reported symptoms and severity scores. The sample size was further reduced because most studies distributed the total number of participants across several intervention groups.

Most of the studies were conducted in both sexes. One study included only men [61], and one included only women [46]. One study included both men and women, but the sex distribution was not reported [76], and one study did not report the sex of the participants studied [72]. In the analyzed 39 studies presented in this review, a total of 2604 participants were examined. Of these, 1296 were women, and 1062 were men. The youngest participants studied were, on average, 24 years old [53], and the oldest participants were, on average, 69 years old [71]. In one study, only the age range (18–45 years) of the participants was provided [47]. Another study reported the average age, but this also included the age of excluded participants who did not complete the training program [50]. Overall, most of the participants were 40–60 years old.

### 3.2. Start of Rehabilitation

Out of 39 studies, 19 reported an average time period between SARS-CoV-2 infection and the start of the rehabilitation program. Summarized analysis showed that the rehabilitation program started at 28.87 ± 12.6 weeks (mean ± SD) after SARS-CoV-2 infection. The minimum, maximum and median were as follows: 5.71, 51.0 and 28.3 weeks after SARS-CoV-2 infection. Categorizing the weeks until the start of rehabilitation results in the following sample sizes for these categories: 0–10 weeks (*n* = 1 study), 10–20 weeks (*n* = 4 studies), 20–30 weeks (*n* = 6 studies), 30–40 weeks (*n* = 4 studies), 40–50 weeks (*n* = 2 studies) and 50–60 weeks (*n* = 2 studies).

Fourteen studies did not report the average time between infection and the start of rehabilitation. Of these, three studies stated that symptoms should persist for at least 4 weeks [48,57,78]. Six studies stated that symptoms should persist for at least 3 months [54,58,63,68,73,81], and three studies stated that symptoms should persist for at least 6 months [47,72,77]. Two of these thirteen studies did not specify a minimum duration of symptoms but rather a maximum duration. In one study, fatigue should not last longer than three months [76], and in another, symptoms should not last longer than 12 months [50].

Six studies did not report the time between infection and the start of the rehabilitation [51,52,60,71,75,80]. However, these included definitions of Long-COVID or descriptions of Long-COVID patients with long-term symptoms and were, thus, included in this analysis.

### 3.3. Categories of Interventions

Studies were analyzed in which physical activity/exercise was an important part of the treatment of Long-COVID patients. Physical exercise as the only intervention was described in 3/39 studies [44,45,46]. In one study, the aerobic group performed physical exercise solely while physical exercise combined with breathing exercise was applied in the respective resistance group [47]. Physical exercise combined with breathing exercises was described in seven studies [48,49,50,51,52,53,54], combined with breathing exercise and pacing in one study [55], and combined with sophrology sessions in another study [56]. Physical exercise, breathing exercise and educational sessions were described in five studies [57,58,59,60,61]. In two studies, physical exercise was combined with educational sessions [62,63]. Multidisciplinary rehabilitation programs were described in seven studies [64,65,66,67,68,69,70,71,72] and most include physical exercise, psychological therapy, occupational therapy and nutritional counseling. Some studies used social integration training, LASER therapy, magnetotherapy [70] and speech therapy [64,66,68,71,72]. Two of the multidisciplinary rehabilitation programs used robotic techniques [71,72]. Seven studies described telerehabilitation programs [73,74,75,76,77,78,79]. Two studies applied pulmonary rehabilitation using VR technology [80,81]. One study used pacing to increase activity levels [82]. The programs integrated a mix of aerobic and resistance training, breathing exercises, functional activities, stretching, balance, mobilization, relaxation and other techniques.

Some rehabilitation programs described to follow guideline recommendations, e.g., National Health Fund (NFZ) [82], Austrian guidelines for outpatient pulmonary rehabilitation [69], WHO guidelines for Support for Rehabilitation: Self-Management after COVID-Related Illness [44,55,75,77,82], American College of Sports Medicine (ACSM) guidelines for chronic obstructive pulmonary disease and cardiovascular disease [44,55] or the European Respiratory Society and American Thoracic Society guidelines [81].

### 3.4. Duration, Frequency and Intensity of Training Sessions

The comparison of studies showed a wide range in the duration of treatment, varying from 2 weeks [54,78] to 12 weeks [45,46,48,51,52,77]. A study duration of 2 to 4 weeks was reported in 15 studies, of 5 to 8 weeks in 11 studies, and of more than 8 weeks in seven publications. In some studies, there was no precise information on the duration of the intervention [50,59,64,70,73,79].

The frequency of training sessions also varied widely. While one program was one hour/week [76], others were carried out daily [78], or the number of training sessions increased over the course of the program [58]. In most studies, the duration of each training session ranged from 30 to 60 min. In addition, one study described a training program consisting of 90 min of aerobic exercise and 30 min strengthening training [63]. It remains unclear whether this information was given for one session or for a week.

Rehabilitation protocols that have been indicated as personalized or individualized refer to the maximum heart rate (20–90% of HRmax) or Borg scale (4–6) for endurance training or were based on one repetition maximum (30–80% 1 RM) or Borg scale (4–5) for resistance training. Endurance training was classified into low-intensity (40–60% HRmax) and high-intensity (60–85% HRmax) training. The Borg scale (0–10 or 6–20) was used in most cases to determine dyspnea, fatigue or the degree of exertion. Intensity of the training sessions was controlled and reported in 24 of 39 analyzed studies as either described targets (% of HRmax or % of 1 RM), an intensity corresponding to 90–100% of heart rate determined by an initial 6-minute walk test (6 MWT) [56], the modified Borg scale [45,62,67,76,77,78,82] or HR reserve [58]. In these studies, training was stopped when the HR reached >80% HRmax or if a value of six (scale up to ten) was given on the Borg scale [76]. Another study used the Borg scale to implement a pacing protocol to adopt the activities of everyday life [82]. In addition to these 24 studies, two studies used HR monitors [46,61] but not to control training. Another five studies monitored blood pressure and/or HR and/or oxygen saturation to control training intensity [54,64,68,77] or exercise tolerance [50].

Some studies indicated that their programs were individualized or personalized but did not describe any of these controlling approaches during the training. In these studies, subjects were treated in individual sessions, and the physiotherapists often decided how to proceed [46,49,54,69,75]. However, this approach was not based on any logic that could be applied to other patients. One study described the program as individualized but only used the Borg scale for training controls [59].

### 3.5. Changes in Long-COVID Symptoms Related to the Training Program

Long-COVID symptoms were measured in 29/39 studies, and the outcomes significantly improved in 28/29 studies [44,45,47,48,49,52,53,54,55,56,57,58,59,61,62,66,67,68,69,71,73,74,76,77,78,79,80,81]. Parameters were not affected by the intervention in one study [46].

The most common symptoms investigated were fatigue, dyspnea, anxiety and depression. The symptoms were identified using these methods: Fatigue Assessment Scale (FAS), Chalder Fatigue Score (CFS-11/CFQ-11), Functional Assessment of Chronic Illness Therapy Fatigue Scale (FACIT), COPD Assessment Test (CAT), Modified Medical Research Council dyspnea scale (mMRC) and hospital anxiety and depression scale (HADS).

The influence of the intervention on the severity of fatigue could be compared in 9/39 studies. The remaining studies either did not assess fatigue or used different questionnaires, which prevented a comparison with other studies. Fatigue improved in 8/9 studies, of which two reported only slight improvements, with moderate fatigue remaining after intervention [45,71]. In two studies, fatigue improved from severe to moderate fatigue [62,77] or to a normal range [52,69]. In 2/8 studies, the values could not be compared because no scores were given for the FSS, and normal cut-off values were used [44,55]. The interventions indicating improved fatigue values applied a training program of 12 weeks with three sessions per week involving individualized training and were categorized as physical exercise, breathing exercise and telerehabilitation.

Dyspnea, another Long-COVID-associated symptom, was measured in 15 studies. In 2/15 studies, there were no significant improvements in dyspnea [46,56]. Although dyspnea was determined in a study using the mMRC, only the number of participants not suffering from dyspnea was determined (score <2) [55]. In one study, dyspnea improved only slightly and remained moderate [74]. In another study, dyspnea was already slight before the intervention and improved to very slight (according to the used categories) [48]. In 8/15 studies, dyspnea improved from moderate to slight [44,49,54,58,67,68,69,77] and from severe to slight dyspnea in 2/15 studies [53,73]. These two studies are characterized by an intervention of 5–7 weeks with 3–5 training sessions per week and were categorized as follows: physical exercise, breathing exercise and telerehabilitation. The two studies without significant changes had a small sample size (*n* = 9 and *n* = 17), and dyspnea was not very severe at baseline, which might explain the poor effect of the intervention on this study parameter.

Anxiety and depression were measured in seven studies. In one study, the statistical significance was not tested [79]. In another study, there were no significant differences in anxiety and depression after the intervention [62]. In 3/7 studies, anxiety and depression scores were already in the normal range before the intervention [48,61,77]. In one study, the values for depression were already in the normal range, but anxiety scores were slightly elevated and then improved to normal levels [80]. In only 1/7 studies, both anxiety and depression were slightly increased and improved with the intervention to no anxiety and depression [68]. The interventions of the two studies that showed the greatest improvements [68,80] were characterized by a length of 3 and 6 weeks, with five training sessions per week, and were categorized as multidisciplinary rehabilitation programs and VR technology. Overall, average scores for anxiety and depression were very low, and to evaluate whether training is beneficial to improve anxiety and depression in Long-COVID, measurements should be performed with respective patients suffering from these symptoms.

The PCFS was only determined in 5/39 studies, and results were either provided as median or mean values. Participants of 1/5 studies had negligible functional limitations [54]. Participants of 2/5 studies showed mild functional limitations at baseline [69,77] that improved to negligible functional limitations. Participants of 1/5 studies showed moderate functional limitations, which turned into negligible functional limitations during treatment [55]. Participants in 1/5 studies had severe functional limitations and only had mild functional limitations after the intervention [68]. The intervention with the greatest improvements in PCFS lasted 8 weeks, with three sessions per week. It included individualized endurance and strength training and fell into the category of physical exercise, breathing exercise and pacing.

### 3.6. Changes in Quality of Life Related to the Interventions

Quality of life (QOL) was measured in 25/39 studies, of which 22 showed significant improvements [44,48,54,55,56,58,59,61,62,64,65,66,67,68,69,71,72,73,74,77,79,82] and three showed no significant improvements [49,51,80]. QOL was measured by the following questionnaires: health-related quality of life (HRQoL), EuroQal 5 domains (EQ5D), EuroQol Questionnaire Visual Analog Scale (EQ VAS), 36-question short-form health survey (SF-36), Barthel index, Patient Health Questionnaire (PHQ-9), Nottingham health profile (NHP), International Classification of Functioning and Disability and Health (ICF). The results from 20 studies were compared because of the use of comparable questionnaires. The most frequently used tests were EQ-5D index (scores: 0–1) (four studies) and VAS (scores: 0–100) (eight studies), SF-12 (PA) and (MH) (average of population: 50) (two studies), Barthel index (scores: 0–100) (four studies) and VQ11 (scores: 11–55) (two studies).

QOL was significantly decreased before the start of the intervention. However, in one study, the values were only [54] slightly above average [83,84]. These participants also showed low functional limitations, low dyspnea and only slight improvements in the 6 MWT (6%), which indicates that this group of participants had only slight limitations before rehabilitation in general and compared to the other studies, which might explain the high improvement score.

Six studies showed improvements in QOL of less than 10%, which could be attributed to normal fluctuations [62,64,71,72,79,82], and eight studies indicated improvements ranging from 10% to 19% [48,54,56,59,61,67,69] or 40% [68] depending on the used questionnaire. Two studies that evaluated the SF-12 found significant improvements, but scores remained below 50 points, which is the US average [44,55]. Despite these improvements, the values remained conspicuous, and the subjects still needed help in everyday life.

The studies that showed the greatest improvements in QOL lasted 3 to 6 weeks with two to seven sessions per week, and the studies were assigned to the following categories: multidisciplinary rehabilitation program, including individual endurance and/or resistance training [67,68,69], physical exercise, breathing exercise and educational sessions [59] or physical exercise with sophrology [56].

### 3.7. Changes in Physical Fitness Related to the Interventions

Physical fitness was measured in 30/39 studies, of which 29 showed significant improvements [44,45,46,47,48,52,53,54,55,56,57,58,61,62,63,64,65,67,69,71,72,73,74,75,76,77,78,80,81], and one showed no significant improvement [51]. Physical fitness was measured using the six-minute walk test (6 MWT), 10 m walk test (10 MWT), sit-to-stand-test (STST), time-up-and-go (TUG), 30 s Chair Stand Test (CST), cardiopulmonary exercise test peak load (CPX peak load), incremental and endurance shuttle walking test (ISWT/ESWT), short performance physical battery test (SPPB), cardiopulmonary exercise test (CPET) and hand grip force (HGF). The tests for physical fitness compared herein were carried out in 25/30 studies. The 6 MWT was carried out in 18/25 studies, HGF was measured in 7/25 studies, and VO_2_max and 1MSTST were in 3/25 studies.

Three of the analyzed 18 studies that used the 6 MWT reported improvements of less than 10%. However, 10/18 studies reported an increase of 10–20% in the 6 MWT [48,51,52,56,57,58,67,69,80,81]. Of these, one study indicated that the values were not significant [51]. The duration of these interventions ranged between 3 and 12 weeks. In 5/18 studies, the improvements in the 6 MWT were in the range of 30–107%. These interventions lasted 2–6 weeks [53,64,65,72,78]. In one study, a distinction was made between mild/moderate and severe/critical COVID diseases [65]. The participants in the mild/moderate groups showed less than 10% improvement, and the severe/critical group showed 36% improvement in the 6 MWT. In two studies, the control groups, who were asked to perform their activities of daily living (ADL), showed no significant changes in the 6 MWT compared to the intervention groups [51,78].

HGF was measured in 7/39 studies [44,45,48,55,63,71,72]. The lowest values measured prior to the start of the rehabilitation were 13.20 kg [72]. In 5/7 studies, HGF improved by 11.11–36.97%, and in 2/7, there were no significant differences [55]. The intervention that showed the greatest improvements [72] lasted 6 weeks, the training sessions were carried out 6 days a week, and individualized endurance training and units on an EMG robot were used. The intervention belonged to the multidisciplinary rehabilitation category.

VO_2_max was measured in 3/30 studies. One study reported no significant improvements [55], one study showed an improvement of more than 10% in the aerobic group (12.96%) [47], while the changes in VO_2_max were <10% in the resistance group (+8.85%) of the same study; and one study showed improvements of <10% in the intervention group [44]. The highest improvements might be related to the applied individualized endurance training, which was carried out five times a week for 4 weeks and belonged to the category of physical exercise.

The 1MSTST was used in 3/39 studies, and all studies indicated an increase in repetitions during the respective intervention [56,69,75].

### 3.8. Changes in Pulmonary Parameters

Pulmonary parameters were measured in 15/39 studies. Improvements were measured in thirteen of them [44,50,53,54,56,57,60,63,64,65,67,69,76], and two showed no significant improvements [48,81].

Arterial oxygen tension (PaO_2_), forced expiratory volume in 1 s, predicted (FEV1%, pred), forced vital capacity, predicted (FVC%, pred), diffusion lung of carbon monoxide FVC, predicted (DLCO%, pred), VO_2_max and VO_2_peak, peak expiratory flow (PEF%, pred) and maximal mid-expiratory flow (MMEF 25–75%, pred) were described to be used in the different studies.

FVC, FEV1 and PaO_2_, which are among the most frequently collected parameters, were recorded in 9/15 studies. One study only provided information on the group effect and not the time effect [44]. The values were already in the normal range before the intervention and hardly changed (less than 1%). In 2/9 studies, only FEV1 was measured [67,69], and in 7/9, both FVC and FEV1 were measured [44,50,53,56,57,60,65]. In 8/9 studies, the values were already within the normal range (>80% or 2–4 L) [85,86] before the start of the intervention. In 1/9 studies, they were slightly below the normal range at 78.86% for FEV1% and 76.32% for FVC% [57].

FEV1 either did not change [56] or FEV1% and FVC% slightly improved by ~7% [57]. Individual FEV1% values improved by more than 10% (10.45–15.7%) during the intervention in three studies [50,56,65]. PaO_2_ was measured in 2/9 studies. In one of these reports, the values were in the normal range (75–100 mmHg) [87] before the start of the intervention [67], and in the other reports, values were slightly below (73.1 mmHg) and improved to the normal range [65].

### 3.9. Adverse Events in the Therapies

A total of 16/39 studies documented whether adverse events occurred during the respective program. No adverse events were identified in 12/16 studies [44,54,55,60,62,65,69,71,73,75,76,77], but 4/16 studies reported adverse events related to the intervention [64,74,79,82]. In one study, adverse events included musculoskeletal pain, dizziness, nausea, hypotension, hypertension and transient chest pain. In addition, non-exercise-related adverse events were reported before initiating physiotherapy sessions. These adverse events were nausea, tachycardia at rest, transient chest pain, infection, dizziness and low blood sugar [64]. None of the respective studies paused the study, but in one study, the intensity was reduced (telerehabilitation program) [74]. One study recorded 6–9% adverse events and 2–5% serious adverse events in the intervention and usual care groups [79]. Twenty-one serious adverse events and 19 concerned admissions to the hospital or prolongation of admission were reported in this study. However, whether these events were related to the intervention was not reported. Only one serious adverse event (syncope with vomiting 24 h after training) was possibly related to the intervention [79]. Adverse events related to the intervention were unilateral knee pain, severe anxiety before the session and headache. There were no cases of exacerbation of symptoms after exercise during weekly monitoring. In one study using pacing protocols, four patients were unable to comply with this guidance because they were unwell with an acute infection separate from COVID-19, a second infection with COVID-19 or an exacerbation of PEM, which significantly limited capabilities [82]. In another study, subjects were told to report any discomfort during the intervention. However, the study did not mention any possible discomfort or adverse events [47].

## 4. Discussion

Due to the high incidence of SARS-CoV-2 infection during the course of the pandemic, a significant proportion of the total population is now affected by Long-COVID. Studies indicate a substantial prevalence of Long-COVID resulting in loss of life quality, health issues and a potential impact on the healthcare system [88,89]. That is why effective therapies are needed to improve the rehabilitation process. However, there is no standardized therapy to treat Long-COVID, although experts favor a holistic form of therapy. Yet, practice shows a heterogeneous picture with regard to treatment concepts.

The aim of this review was to analyze studies that present possible recent treatment strategies that include physical exercise because of its known benefits during the recovery of various diseases [29,90]. The results were critically analyzed according to the following categories: training modalities (duration of intervention, frequency and intensity of training session) and a possible influence of these factors on Long-COVID symptoms, quality of life, physical fitness and pulmonary parameters.

### 4.1. General Observations in the Comparison of the Studies

The sample sizes of the analyzed studies were rather small, with the exception of one trial that included more than 100 subjects. This is important to mention because a small overall sample size might limit the significance, accuracy and representativity of the results, especially in light of the heterogeneity of Long-COVID disease. A large sample size also minimizes distortions in the results.

Another factor that showed a high degree of heterogeneity between the studies was the period between the SARS-CoV-2 infection and the start of rehabilitation, ranging between 4 weeks and >6 months after SARS-CoV-2 infection. However, all studies reported positive outcomes and no differences in the magnitude of the changes could be detected. Thus, the start of early rehabilitation might positively affect the overall course of the disease. Yet, positive changes can be achieved through intervention even after a long period of illness, so intervention might be beneficial for rehabilitation at any time.

The analyzed studies also differed greatly in length, duration and intensity of the conducted training sessions. The length of the interventions ranged between two and 12 weeks, the frequency varied between one and six sessions per week, and intensities of the training sessions were either not reported or, when reported, ranged from moderate intensities (40–60% of HRmax) to high intensities (60–85% HRmax or 30–80% of 1 RM). The combination of these factors seems to be crucial for the effectiveness of the conducted therapy. Long-lasting interventions might be more promising compared to shorter durations, given the heterogeneity and individual course of the disease and the fact that Long-COVID-related symptoms, QOL and fitness level of the analyzed studies remained below average. Not only the length of the intervention might affect the outcome, but also the number of training sessions completed during that time. For example, Binetti et al. used a protocol for 3 months, but only 12–20 sessions were carried out during that time [46], suggesting that in some cases, only one session per week was performed. The respective study showed no significant improvements in Long-COVID symptoms or physical fitness, indicating that the length, duration and frequency of the treatment did not match the needs of the tested subjects well. In contrast, a study that lasted only four weeks and included six training sessions/week showed significant improvements in Long-COVID symptoms, physical fitness and pulmonary parameters [53]. However, it should be noted that this study examined the youngest participants and that age may also be a factor in evaluating the effectiveness of interventions in Long-COVID.

In order to avoid post-exertional malaise and to support optimal recovery between sessions, the frequency of the training sessions should not be too high, and the intensity should be balanced between adequate training stimulus and preventing the worsening of Long-COVID symptoms. The monitoring of PEM was underrepresented in the analyzed studies, although it is well known that overexertion favors PEM and worsens Long-COVID symptoms [91] and the rehabilitation process. Because PEM can occur frequently, monitoring and classification of respective PEM events is necessary and would require [92] adjustment of training stimulus.

The consideration of Long-COVID severity was also underrepresented and only evaluated in five of the 39 analyzed studies. However, assessment of Long-COVID severity, e.g., using the Bahmer score [6], but also considering pre-existing co-morbidities would be important to assess the overall condition of the participants to be recognized during the training and also for the conceptualization of the training program.

### 4.2. Exercise-Based Therapy Improves Long-COVID Symptoms, Quality of Life, Pulmonary Parameters and Physical Fitness

#### 4.2.1. Improvements in Long-COVID Symptoms

Fatigue, dyspnea and anxiety/depression are common symptoms in Long-COVID [93,94], and it is, therefore, useful to assess these changes related to an exercise intervention. The presented results showed a high degree of heterogeneity possibly associated with the conceptualization of the training program, the used survey methods and the Long-COVID severity of the tested subjects. The largest changes in fatigue scores, pulmonary parameters and anxiety/depression were observed in studies with a training duration > 5 weeks and 3–5 training sessions per week. However, the severity of the various symptoms should be determined because it became apparent in this review that some baseline levels, e.g., for anxiety/depression and pulmonary parameters, were inconspicuous. These results suggest that the subjects might have had a lower level of severity of Long-COVID and that the concept may not be applicable to Long-COVID patients with severe limitations. The results of the analyzed studies further indicate that even after 12 weeks of training, Long-COVID-related symptoms remain striking. A program with no time limit could accompany patients until they are fully recovered.

#### 4.2.2. Improvements in Quality of Life

The quality of life of Long-COVID patients is severely limited, which includes health-related QOL that might be related to fatigue [95]. The reduction in QOL is confirmed by the analyzed studies. The training program of the studies compared here highlights the beneficial effect of training on QOL. The interventions with the greatest improvements lasted for 3–6 weeks with 2–5 sessions/week. However, the need for assistance with ADLs remained after the training program. This suggests that the duration of the interventions was not sufficient to improve the QOL of all participants to the point where they could perform ADLs independently.

#### 4.2.3. Improvements in Physical Fitness

Physical fitness has been reported to be impaired in Long-COVID [96] and has been linked to poor QOL and severity of fatigue [97].

Parameters related to physical fitness improved, with the greatest changes observed at 3–12 weeks and five sessions/week. Two studies showed a rather small effect of the intervention on the physical fitness of the participants. However, the sample size of these studies was very small (*n* = 9 and *n* = 15), which might have affected the outcome, given the great heterogeneity of the Long-COVID patients [43,61]. Further, the duration of the interventions was rather short (2 and 4 weeks), which might explain the small changes [61,62].

Thus, exercise has a greater effect on physical fitness in Long-COVID patients than ADLs. Physical fitness was often determined using the 6 MWT, but the analysis of the studies revealed that only one study provided reference values regarding distance that needs to be achieved during 6 MWT. The Trooster or Enright and Sherrill formula can be used to calculate the target distance for the 6 MWT [98,99]. These formulas consider height, weight, age and gender because these factors influence the walking distance. No such differentiation or comparison with standard or target values was carried out in any of the studies. However, an age- or sex-specific analysis is needed for the comparison of fitness tests. This also applies to the HGF, VO_2_max or 1MSTST [100,101].

It should also be noted that when fitness tests are repeated, results are often better on the second test than on the first. The reasons for this are assumed to be as follows: a training effect, a more realistic assessment of one’s own performance and less anxiety when assessing individual resilience. In addition, it is important to assess the results not only in relation to a previous comparison but also in relation to standard/reference values. This is because it is not only important to know whether scores have improved but also to compare the test results of ill people with those of healthy people.

### 4.3. Limitations

The literature search was restricted to the databases Pubmed, PEDro, BioMed Central, EBSCOhost, ProQuest and the ZBSport, and the literature search was also restricted to English language publications. Therefore, it cannot be guaranteed that there may be other studies on this topic that are not included in this review. The risk of bias in the studies was only assessed in approach and not compared with each other as the different study designs did not allow for a standardized comparison. Limitations of the included studies were also identified. When searching the literature, we noticed that various definitions and terms are used for Long-COVID. In order to ensure that the participants studied were actually Long-COVID patients, the duration between infection and start of intervention was determined, or the existence of a corresponding definition was assumed. This was necessary because there were too few studies that specified a Long-COVID diagnosis as an inclusion criterion (only two studies) [46,75]. A Long-COVID diagnosis (U09.9) as an inclusion criterion would be important for future studies to ensure that they are indeed Long-COVID patients.

Rehabilitation programs are usually limited in time. This review showed that the programs lasted between 2 and 12 weeks. During this time, improvements in the various examined parameters (symptoms, quality of life, physical fitness, pulmonary parameters) were identified. However, it was not always sufficient to normalize values, especially with regard to the symptoms of fatigue and quality of life. Therefore, no recommendations for the length of interventions could be given. However, it shows that there is a need for continued care of Long-COVID patients. In the future, time-unlimited programs should be developed to ensure continued care even after rehabilitation.

In some studies, the sample size was very small, or participants were also divided into different intervention groups. These studies may, therefore, provide imprecise or unrepresentative results. Further studies with a larger sample size are therefore necessary in order to improve and substantiate the validity of the results.

It was not possible to rank the interventions in terms of effectiveness, even for the different effects (symptoms, quality of life, physical fitness, pulmonary parameters), as the studies were so different in other aspects (high heterogeneity).

Overall, the comparison was very difficult because the same parameters were examined using a wide variety of measurement methods. There is no uniform procedure and no standard for Long-COVID treatment and its measurements. This is, of course, also due to the fact that this is a relatively new disease. However, it is therefore necessary to develop specific tests for Long-COVID. These can then be used in future studies to make them more comparable in terms of their effects.

## 5. Conclusions

The comparison of the studies evidenced that supervised exercise training might represent an effective therapy method for patients suffering from Long-COVID because of its positive effects on Long-COVID-related symptoms, QOL and physical fitness. However, the comparison of analyzed studies showed a high heterogeneity regarding the following factors: sample size, sex-specific analysis, the severity of Long-COVID disease, training modalities (content, length, duration, intensity, frequency), the measuring method applied or the reference values considered. Furthermore, the results indicate that programs combining physical training (aerobic and strength training) with other therapeutic approaches, such as respiratory training, pacing or physiotherapy, for example, achieved the most significant progress regarding the analyzed categories: Long-COVID symptoms, physical fitness, respiratory parameters and quality of life. These programs were typically interdisciplinary in nature, with a duration of >5 weeks and three individualized training sessions per week.

It is important to note that some of the Long-COVID patients tested in the studies appear to continue to suffer from Long-COVID after the respective intervention, as the described post-intervention scores for physical fitness, QOL and Long-COVID symptoms were outside the normal range.

Future research should aim to develop and refine interdisciplinary rehabilitation concepts. These concepts should be based on a structured, long-term and consecutive treatment approach and ideally starting soon after the diagnosis of Long-COVID, as this may be beneficial for the course of the disease. When designing the individualized program, it is important to take into account the severity of the condition and individual symptoms, as well as other physical limitations and the physical fitness of the patient. In particular, individual Long-COVID symptoms, such as exercise intolerance and post-exertional malaise (PEM), should be taken into account when managing exercise. Given the uncertainties identified regarding the timeframe required for significant improvements in these areas, it seems crucial to adopt a time-independent treatment approach tailored to the individual needs of patients and their recovery process. This will help prevent a deterioration of their condition and provide optimal support and care for the patients.

## Figures and Tables

**Table 1:** Overview of included articles presenting participants, interventions and effects.

Author, Year	Participants: Number, Age, Sex (f/m)	Intervention	Duration, Frequency, Dose	Effect (Improvements: yes/no/n. m.)	Quality Appraisal and Scoring ^1^
**PHYSICAL EXERCISE**					
Jimeno-Almaz et al., 2022, [44]	39 45.2 ± 9.5 (29/10)	IG: Concurrent training (resistance training combined with aerobic training) CG: followed WHO guidelines for rehabilitation after COVID-19 (aerobic and strength exercises)	8 weeks 2 sessions/week 50% 1 RM 55–65% HRR, 65–70% HRR and 70–80% HRR Used RPE and individual progression	Symptoms: yes Quality of life: yes Physical fitness: yes Pulmonary parameters: yes No adverse events	Score 4 out of 4 (high)
Mohammed et al., 2023, [45]	54 (19/35)	Thai Chi group Aerobic training group ADL group	12 weeks 4 sessions/week 50–70% HRmax and 40–60% HR max Borg scale (4–6/10)	Symptoms: yes Quality of life: n. m. Physical fitness: yes Pulmonary parameters: n. m.	Score 4 out of 4 (high)
Binetti et al., 2023, [46]	9 44.21 Only female	Physiotherapy: stretching, aerobic exercise, strengthening exercise	3 months 12–20 sessions Progression in duration and intensity HR monitoring	Symptoms: no Quality of life: n. m. Physical fitness: yes Pulmonary parameters: n. m.	Score 2 out of 4 (poor)
**PHYSICAL EXERCISE AND BREATHING EXERCISE **					
Azizbhai et al., 2023, [47]	34 18–45 years (23/11)	Resistance group Aerobic group	4 weeks Aerobic: 5 days/week and resistance: 2 days/week 50–60% HRR and used RPE (4–6/10) 60–80% of 1 RM	Symptoms: yes Quality of life: n. m. Physical fitness: yes Pulmonary parameters: n. m.	Score 3 out of 4 (moderate)
Dierckx et al., 2024, [48]	17 42 ± 13 (12/5)	Endurance, resistance and strength training, inspiratory muscle training	3 months 3 sessions/week Cycling: 50% of the load reached at anaerobic threshold Treadmill: 60% walking speed, 60% of 1 RM, 60% of MIP	Symptoms: yes Quality of life: yes Physical fitness: yes Pulmonary parameters: no	Score 2 out of 4 (poor)
Romanet et al., 2023, [49]	60 (23/37)	IG: endurance and strength training CG: physiotherapy: aerobic and strength training, stretching, balance, electrostimulation, respiratory therapy	90 days/10 weeks 2 sessions/week IG: 60 min and CG: 30 min Endurance: 60–70% of maximal peak power Used Borg scale for dyspnea (4–6/10) and muscle fatigue	Symptoms: yes Quality of life: no Physical fitness: n. m. Pulmonary parameters: n. m.	Score 3 out of 4 (moderate)
Mińko et al., 2023, [50]	150 64.66 ± 11.93	Aerobic training, strength training, march training, endurance exercises	2–6 weeks 6 days/week all types of training 30 min 60–85% of 1 RM with progression Used Borg Scale (2–3/10) and in exercise tolerance	Symptoms: n. m. Quality of life: n. m. Physical fitness: n. m. Pulmonary parameters: yes	Score 3 out of 4 (moderate)
Oliveira et al., 2023, [51]	59 52.32 + 11.87 (34/25)	IG: mobility, stretching, breathing techniques, resistance, strength, balance and relaxation CG: no training but received educational orientation and performed ADLs	12 weeks 24 sessions 2 sessions/week 60 min	Symptoms: n. m. Quality of life: no Physical fitness: no Pulmonary parameters: n. m.	Score 4 out of 4 (high)
Ali et al., 2023, [52]	60 45.7 ± 2.40 (31/29)	Group A: physiotherapy: aerobic exercise, muscle strengthening and respiratory exercise Group B: active cycle of breathing technique and physiotherapy (see Group A)	12 weeks 3 sessions/week 30–65 min 30–40% 1 RM to 80% 1 RM HR, oxygen serration and Borg Sale	Symptoms: yes Quality of life: n. m. Physical fitness: yes Pulmonary parameters: n. m.	Score 4 out of 4 (high)
Sanchez-Mila et al., 2023, [53]	200 (100/100)	IG: inspiratory muscle training with device and aerobic exercise CG: respiratory/diaphragmatic exercises and aerobic exercise	31 days 6 sessions/week 60–75% HRmax and 50–60% VO_2_max	Symptoms: yes Quality of life: n. m. Physical fitness: yes Pulmonary parameters: yes	Score 4 out of 4 (high)
Szarvas et al., 2023, [54]	68 53.5 (29/39)	Breathing techniques, chest mobility, muscle strengthening Aerobic respiratory muscle strengthening, stretching, active cycle breathing	2 weeks 2–3 sessions/week 30 min Monitoring of HR and oxygen saturation 40% MIP	Symptoms: yes Quality of life: yes Physical fitness: yes Pulmonary parameters: yes No adverse events	Score 3 out of 4 (moderate)
**PHYSICAL EXERCISE, BREATHING EXERCISE AND PACING**					
Jimeno-Almaz et al., 2023, [55]	80 45.3 ± 8.0 (55/25)	Concurrent training group: resistance and endurance Inspiratory muscle training group: combination group: CG: self-management WHO	8 weeks 3 sessions/week 50% 1 RM, 55–65% HRR and 70–80 HRR, ~60% MIP Used RPE: resistance, aerobic and inspiratory muscle training	Symptoms: yes Quality of life: yes Physical fitness: yes Pulmonary parameters: n.m. No adverse events	Score 4 out of 4 (high)
**PHYSICAL EXERCISE AND SOPHROLOGY**					
Vallier et al., 2023, [56]	17 54.8 ± 16.0 (5/12)	Endurance sessions, gymnastics/muscular strength, sonography, medical consultation (dietician, psychologist, physician before and after rehabilitation)	4 weeks 7 sessions/week (4 times walks and 3 times gymnastics) At 90–100% HR achieved at the end of 6 MWT	Symptoms: yes Quality of life: yes Physical fitness: yes Pulmonary parameters: yes	Score 3 out of 4 (moderate)
**PHYSICAL EXERCISE, BREATHING EXERCISE AND EDUCATIONAL SESSIONS**					
Ponce-Campos, et al., 2022, [57]	42 53.35 (17/25)	Physiotherapy: breathing exercises, mobilization, relaxation, progressive strengthening exercise, teaching energy saving techniques, aerobic exercises, balance and coordination	4 weeks 3 sessions/week Individualized according to the need of each patient 55–60%, 60–65% and 70–75% of HRmax	Symptoms: yes Quality of life: n. m. Physical fitness: yes Pulmonary parameters: yes	Score 3 out of 4 (moderate)
Rzepka-Cholasińska et al., 2024, [58]	90 61.65 ± 5.39 (49/41)	Aerobic exercises, strength and resistance training, balance, breathing, respiratory exercises, stretching, education	6 weeks 3 sessions/week 30 min/session Progression 30–60% HRR	Symptoms: yes Quality of life: yes Physical fitness: yes Pulmonary parameters: n. m.	Score 3 out of 4 (moderate)
Mammi et al. 2023, [59]	50 53 ± 11.4 (29/21)	Manual physical therapy, soft tissue, kinesiotaping, stretching, flexibility and mobility, core stability, balance, endurance, educational session	2 sessions/week 10 individual sessions for 45 min Used Borg Scale (for progression)	Symptoms: yes Quality of life: yes Physical fitness: n. m. Pulmonary parameters: n. m.	Score 2 out of 4 (poor)
Pietranis et al., 2024, [60]	59 63.1±13.41 (37/22)	Both groups: physiotherapeutic interventions: aerobic, respiratory and resistance training, overall fitness exercises, stretching, educational sessions IG: resistance training with respiratory muscle trainer CG: placebo respiratory muscle trainer	6 weeks 45–55% to 70–80% HRmax	Symptoms: n. m. Quality of life: n. m. Physical fitness: n. m. Pulmonary parameters: yes No serious adverse events	Score 4 out of 4 (high)
Benzarti et al., 2022, [61]	14 61 ± 4 Only male	Aerobic cycle endurance, strength training, balance, stretching, relaxation, breathing techniques and education	4 weeks 3 sessions/week, 70 min HR monitoring, target HR is HR in the end of 6 MWT	Symptoms: yes Quality of life: yes Physical fitness: yes Pulmonary parameters: n. m.	Score 1 out of 4 (very poor)
**PHYSICAL EXERCISE AND EDUCATIONAL SESSIONS**					
Daynes et al., 2021, [62]	30 58 (14/16)	Aerobic exercise, strength training, educational discussions	6 weeks 2 sessions/week Borg breathlessness scale and rate of perceived exertion were used for progression	Symptoms: yes Quality of life: yes Physical fitness: yes Pulmonary parameters: n. m. No serious adverse events	Score 3 out of 4 (moderate)
Colas et al., 2023, [63]	38 46.9 ± 12.7 (21/17)	Aerobic exercise, resistance exercise, educational sessions	4 weeks 3 sessions/week each for 2 h 2 sessions/week: aerobic exercise 90 min, resistance exercise 30 min	Symptoms: n. m. Quality of life: n. m. Physical fitness: yes Pulmonary parameters: yes	Score 3 out of 4 (moderate)
**MULTIDISCIPLINARY REHABILITATION PROGRAM**					
Halvorsen et al., 2024, [64]	20 62.35 ± 14.02 (11/9)	HIT (endurance), respiratory training, occupational therapy, speech and language pathology	Median 10 sessions in total 5 sessions/week HIT: 4 sessions/week, 60 min/session 70–85% of the age-predicted HRmax Used RPE (14–17/20), monitoring of HR and oxygen saturation	Symptoms: n. m. Quality of life: yes Physical fitness: yes Pulmonary parameters: yes Some adverse events	Score 2 out of 4 (poor)
Gloeckl et al., 2021, [65]	50 (28/22)	Endurance, strength and ADL training, relaxation, respiratory physiotherapy, occupational therapy, psychological and nutritional support, education	3 weeks 5 days/week 60–70% of peak work rate	Symptoms: n. m. Quality of life: yes Physical fitness: yes Pulmonary Parameters: yes No adverse events	Score 3 out of 4 (moderate)
Hasting et al., 2023, [66]	33 49.2 ± 9.8 (24/9)	Neuropsychological treatment, pacing, relaxation and mindfulness techniques, cognitive training, speech and language therapy, physiotherapy (strength, fitness training, mobilization, stretching, breathing exercises), psychoeducation, nutritional counseling, social and medical consultation	3 weeks 10 treatment days: 3–4 units/day Physiotherapy: using pacing to adopt optimal activity rhythm (light to moderate intensity)	Symptoms: yes Quality of life: yes Physical fitness: n. m. Pulmonary parameters: n. m.	Score 2 out of 4 (poor)
Hayden et al., 2021, [67]	53 (28/25)	Physical training (endurance and strength training, vibration training, inspiratory muscle training), respiratory physiotherapy, general physiotherapy, occupational therapy, education, medical diagnostics, psychological and nutritional support	3 weeks 7 days/week 19–21 sessions/week 21–60 min Intensity based on initial 6 MWT Used RPE (4–6/10), oxygen saturation ≥90%, HR monitoring	Symptoms: yes Quality of life: yes Physical fitness: yes Pulmonary parameters: yes	Score 3 out of 4 (moderate)
Kesikburun et al., 2023, [68]	39 59.7 ± 15.6 (15/24)	Mobilization, progressive muscle strengthening, balance and coordination, FES cycling, cardiopulmonary rehabilitation: aerobic exercise, muscle strengthening, breathing exercise, speech, language and occupational therapy, psychological and nutritional support	6 weeks 30 sessions 5 days/week, 1 h/session Monitoring of blood pressure, HR and oxygen saturation in each session	Symptoms: yes Quality of life: yes Physical fitness: n. m. Pulmonary parameters: n. m.	Score 2 out of 4 (poor)
Nopp et al., 2022, [69]	58 47 (25/33)	Individualized endurance, strength and inspiratory muscle training Education, psychosocial counseling, nutritional education, smoking cessation sessions	6 weeks 3 sessions/week, 3–4 h each	Symptoms: yes Quality of life: yes Physical fitness: yes Pulmonary parameters: yes No adverse events	Score 3 out of 4 (moderate)
Grishechkina et al., 2023, [70]	113 58.4 (83/30)	IG: aquatic, respiratory and motor exercises, social integration training, neuropsychologic sessions, LASER therapy, magnetotherapy CG 1: eastern medicine techniques CG 2: respiratory and motor exercise therapy, physiotherapy combined with inhalation, balneo- and magnetotherapy CG 3: self-training and home-based physical exercise	IG: 7–8 sessions CG 1: 7–8 sessions CG 2: 10–15 sessions CG 3: ??	CGs: more hospital admissions, need for specialist consultations and ambulance calls	Score 2 out of 4 (poor)
**MULTIDISCIPLINARY REHABILITATION PROGRAM (ROBOT)**					
Zasadzka et al., 2022, [71]	28 (10/18)	Neuromuscular re-education techniques, coordination, balance, progressive endurance training, psychologist, speech and occupational therapy IG: EMG-rehabilitation robot	6 weeks 6 days/week IG: 75 min, CG: 120 min Progressive endurance training: 30 min, 35–70% HRmax IG: EMG-rehabilitation robot (6 days/week, 45 min/day in 2 sessions)	Symptoms: yes Quality of life: yes Physical fitness: yes Pulmonary parameters: n. m. No adverse events with EMG-rehabilitation robot	Score 4 out of 4 (high)
Trzmiel et al., 2023, [72]	81	Neuromuscular re-education techniques, coordination, balance, progressive endurance training, psychologist, speech and occupational therapy IG: EMG-rehabilitation robot	6 weeks 6 days/week IG: 75 min, CG: 120 min Progressive endurance training: 30 min, 35–70% HRmax IG: EMG-rehabilitation robot (6 days/week, 45 min/day in 2 sessions)	Symptoms: n. m. Quality of life: yes Physical fitness: yes Pulmonary parameters: n. m.	Score 4 out of 4 (high)
**TELEREHABILITATION**					
Calvo-Paniagua et al., 2022, [73]	68 48.5 ± 9.7 (42/26)	Posture ergonomics, respiratory control, physical exercise, aerobic exercise, mobilization, motor control exercise, occupational therapy, diaphragmatic respiratory education	Up to 7 weeks 3 sessions/week, 40 min Physical conditioning with increasing intensity	Symptoms: yes Quality of life: yes Physical fitness: yes Pulmonary parameters: n. m. No adverse events	Score 3 out of 4 (moderate)
Bileviciute-Ljungar et al., 2024, [74]	67 43 (52/15)	Breathing exercise, mindfulness, relaxation, muscle strength training, exercise on one’s own, psychoeducation	8 weeks 3 days/week for 2 h 3 h exercise on one’s own	Symptoms: yes Quality of life: yes Physical fitness: yes Pulmonary parameters: n. m. Some reported a worsening of symptoms	Score 3 out of 4 (moderate)
Estebanez-Pérez et al., 2022, [75]	32 45.93 (23/9)	Physiotherapy: patient education, aerobic exercise, strength and training exercises, breathing exercises, recommendations for secretion drainage and ventilatory techniques	4 weeks Limit: one session/day, 45–50 min Progression of intensity and duration depends on sensation of fatigue and/or dyspnea (strength training: 5–10%/week)	Symptoms: n. m. Quality of life: n. m. Physical fitness: yes Pulmonary parameters: n. m. No adverse events	Score 3 out of 4 (moderate)
Colas et al., 2022, [76]	15 52.1± 12.2 Both sexes	IG: aerobic exercise, resistance exercise, therapeutic education workshops, psychological and nutritional support CG: physiotherapy: delivery of a training booklet with test results of the initial evaluation, psychological and nutritional support	4 weeks 3 sessions/week Used RPE (2–6/10 for), monitoring of HR and RPE training stopped when HR >80% HRmax or RPE >6/10	Symptoms: yes Quality of life: n. m. Physical fitness: yes Pulmonary parameters: yes No adverse events	Score 2 out of 4 (poor)
Reis et al., 2023, [77]	49 (26/23)	IG: ventilatory control training, aerobic exercise, muscle strengthening exercise, respiratory muscle training, flexibility, balance CG: usual care: initial clinical evaluation, management of the therapeutic regimen, education and training relative to their health status	12 weeks 2–3 sessions/week (38 sessions of 60 min) Intensity was adapted according to the perception of dyspnea according to the modified Borg scale (RPE 4–5/10) Monitoring of HR and oxygen saturation	Symptoms: yes Quality of life: yes Physical fitness: yes Pulmonary parameters: n. m. No adverse events	Score 3 out of 4 (moderate)
Rodriguz-Blanco et al., 2023, [78]	48 (26/22)	IG: therapeutic exercise program CG: relative home rest consisting of ADLs	14 days 1 session/day Used modified Borg scale for intensity	Symptoms: yes Quality of life: n. m. Physical fitness: yes Pulmonary parameters: n. m.	Score 4 out of 4 (high)
McGregor et al., 2024, [79]	585 56 ± 12 (305/280)	IG: group exercise, psychological support, on-demand library of physical activity (circuit, Interval, aerobic, breathing exercise, Pilates, yoga) CG: usual care: best practice usual care consisting of online, one-to-one consultation with a trained practitioner	1–8 weeks 3–4 sessions/week, 30–60 min CG: one session 30 min	Symptoms: yes Quality of life: yes Physical fitness: n. m. Pulmonary parameters: n. m. Several and serious adverse events	Score 3 out of 4 (moderate)
**VR TECHNOLOGY**					
Rutkowski et al., 2022, [80]	32 57.8 ± 4.9 (20/12)	Exercise capacity, walking and resistance training, general fitness exercise, circuit training, breathing exercise, relaxation, techniques for removing secretions from the bronchial tree, inhalations VR group: cycling and relaxation with VR googles	3 weeks 5 sessions/week Cycling: intensity based on patient’s submaximal exercise tolerance test results 20–80% of the submaximal HR	Symptoms: yes Quality of life: no Physical fitness: yes Pulmonary parameters: n. m.	Score 4 out of 4 (high)
Rutkowski et al., 2023, [81]	32 57.8 ± 4.92 (20/12)	Exercise capacity training, walking training, resistance training, general fitness exercise, circuit training, breathing exercise, relaxation, techniques for removing secretions from the bronchial tree, inhalations VR group: cycling and relaxation with VR googles	3 weeks 5 sessions/week Cycling: intensity based on patient’s distance and dyspnea rating in 6 MWT 20–90% of HR peak	Symptoms: yes Quality of life: n. m. Physical fitness: yes Pulmonary parameters: no	Score 4 out of 4 (high)
**PACING**					
Parker et al., 2023, [82]	31 47 ± 9 (22/9)	WHO Borg CR-10 pacing protocol for physical activity guidance	6 weeks Using WHO Borg CR-10 pacing protocol Used RPE for progression	Symptoms: n. m. Quality of life: yes Physical fitness: n. m. Pulmonary parameters: n. m. Less average number of PESE episodes	Score 2 out of 4 (poor)

f, female; m, male; n. m. not measured; HRR, heart rate reserve, HRmax, maximal heart rate; HRpeak, peak heart rate; IG, intervention group; CG, control group; MIP, maximal inspiratory pressure; ADLs, activities of daily living; 6 MWT, 6-minute walk test; CR-10 Borg scale, Category-Ratio-10 Borg scale; RPE, Borg Rating of Perceived Exertion scale; 1 RM, one repetition maximum; HIIT, high intensity interval training; HIT, high intensity training; PESE, Post-Exertional Symptom Exacerbation; FES cycling, functional electrical stimulation cycling. ^1^ (1) sample size: ≥ 30 = 1; smaller sample = 0; (2) randomized controlled trials = 1; not randomized, no controls = 0; (3) studied one or more categories (symptoms, quality of life, physical fitness, pulmonary parameters) = 1; otherwise = 0; (4) standard and objective evaluation criteria = 1; otherwise = 0.

## Supplementary Materials

The following supporting information can be downloaded at: https://www.mdpi.com/article/10.3390/diseases12110293/s1, Table S1: Detailed overview of included articles presenting participants, interventions and effects.
