# Supplementary material for: Identifying Factors That Might Affect Outcomes of Exercise-Based Therapies in Long-COVID
Source: Diseases. 2024 Nov 15;12(11):293. doi: 10.3390/diseases12110293 (PMC11593325; doi:10.3390/diseases12110293)
Supplement: Supplementary file 1 [file diseases-12-00293-s001.zip › diseases-3213760-supplementary.pdf]

Table S1. Detailed overview of included articles presenting participants, interventions and effects.

Author, year	Participants: number, age, sex (f/m)	Intervention	Duration, frequency, dose	Effect [improvements: yes/no/n. m.]
**PHYSICAL EXERCISE**				
Jimeno-Almaz et al., 2022, [44]	39<br>45.2±9.5<br>(29/10)<br>IG: 20<br>CG: 19	IG: Concurrent training (resistance training combined with aerobic training)<br>CG: followed WHO guidelines for rehabilitation after COVID-19 (aerobic and strength exercises)	8 weeks<br>IG: resistance training 2 sessions/week 3 sets, 8 rep., 4 exercises with 50% 1RM combined with moderate intensity variable training (4-6 x 3-5 min at 70-80% HRR/2-3 min at 55-65% HRR), RPE not higher than 16<br>1 day/week light intensity continuous training (30-60 min, 65-70% HRR), RPE 11-12, individual progression (patient tolerance)<br>CG: aerobic exercise: 5 days/week 20-30 min intensity that allows breathless speech; strength exercise: 3 days/week 7 recommended exercises 3 sets 10 rep.	Symptoms: yes<br>Quality of life: yes<br>Physical fitness: yes<br>Pulmonary parameters: yes<br>No adverse events
Mohammed et al., 2023, [45]	54<br>(19/35)<br>Thai Chi: 18, 65.7±3.6, (6/12)<br>Aerobic training: 18, 66.2±3.8, (7/11)<br>CG: 18, 66.3±4, (6/12)	Thai Chi: warm up, Thai Chi (7 movements), relaxation<br>Aerobic training: stretching, upper and lower limbs muscle training, treadmill walking<br>CG: usual ADLs	12 weeks<br>Thai Chi: 4 sessions/week 60 min, 50-70% HRmax or 4-6 Borg scale (40 min Thai Chi: 4 rep. of movements)<br>Aerobic training: 4 sessions/week 60 min, 40-60% HRmax or 4-6 Borg scale (20 min strengthening with 0.5 kg weight, 15-20 min treadmill walking 1.2-2.6 km/h)<br>CG: maintain a daily routine	Symptoms: yes<br>Quality of life: n. m.<br>Physical fitness: yes<br>Pulmonary parameters: n. m.
Binetti et al., 2023, [46]	9<br>44.21<br>Only female	physiotherapy sessions: stretching, aerobic exercise, strengthening exercise	3 months<br>12-20 sessions<br>stretching: 5-10 min,<br>aerobic exercise: 10-30 min, with progressive duration, intensity according to effort tolerance, with HR monitoring<br>strengthening exercise: in one of the weekly sessions, for upper limbs,<br>Aerobic routine: (walking) on the days without sessions	Symptoms: no<br>Quality of life: n. m.<br>Physical fitness: yes<br>Pulmonary parameters: n. m.
**PHYSICAL EXERCISE AND BREATHING EXERCISE**				
Azizbhai et al., 2023, [47]	34<br>18-45 years<br>(23/11)<br>Aerobic group: 17<br>Resistance group: 17	Resistance group: Warm up (aerobic 10 min, resistance 5 min): mobilization<br>Aerobic group: Brisk Walking<br>resistance group: all major muscle groups of upper and lower limbs, diaphragmatic exercise, purse lip breathing exercise<br>Cool down: 10 min stretching	4 weeks<br>Aerobic: 5 days/week, 50-60% HRR, or 4-6 RPE, 20 min with progression with 5-10 min/day once or twice a week, speed 3.5 km/hr<br>Resistance: 2 days/week, 60-80% of 1RM, 2-4 sets with 8-12 rep., with progression in rep. and sets	Symptoms: yes<br>Quality of life: n. m.<br>Physical fitness: yes<br>Pulmonary parameters: n. m.
Dierckx et al., 2024, [48]	17<br>42±13<br>(12/5)	Personalized pulmonary rehabilitation program<br>Endurance: cycling, treadmill walking, arm exercise<br>resistance and strength training:<br>inspiratory muscle training: threshold device	3 months<br>3 sessions/week<br>Cardio exercise: 10 min cycling: 50% of the load reached at the anaerobic threshold; treadmill: 60% walking speed<br>Strength training: 60% of 1RM 8 rep.<br>Inspiratory muscle training: 60% of MIP, progression	Symptoms: yes<br>Quality of life: yes<br>Physical fitness: yes<br>Pulmonary parameters: no
Romanet et al., 2023, [49]	60<br>(23/37)	IG: endurance (cycle ergometer) and strength training (lower and upper limbs, core)	90 days/10 weeks<br>IG: 2 sessions/week, 60 min	Symptoms: yes<br>Quality of life: no

	IG: 27, 57, (11/16)<br>CG: 33, 59, (12/21)	CG: Standard physiotherapy (low-moderate intensity aerobic training, strength training, stretching, balance, electrostimulation, respiratory therapy)	Endurance: 60-70% of maximal peak power, 4-6 Borg scale for dyspnea<br>strength: 4 sets 6-12 rep.<br>CG: 2 sessions/week, 30 min<br>both: muscle fatigue had to be felt at the end of each set	Physical fitness: n. m.<br>Pulmonary parameters: n. m.
Mińko et al., 2023, [50]	150<br>64.66±11.93	Rehabilitation program: Aerobic training, strength training, march training, endurance exercises	2-6 weeks<br>6 days/week<br>General fitness and respiratory improvement exercises: 30 min (breathing exercises)<br>Aerobic training: 30 min (2-3 Borg scale)<br>Continuous/interval endurance training: 30 min, low or moderate intensity, gradual increase by 5-10% (2-3 Borg scale)<br>Outdoor march training: 2 sessions/day each for 30 min (2-3 Borg scale)<br>Strength and endurance training: 30 min (70-85% of 1RM, 3 sets 8-12 rep.), progression: 60-70% of 1RM<br>all sessions: in exercise tolerance (no presence of desaturation)	Symptoms: n. m.<br>Quality of life: n. m.<br>Physical fitness: n. m.<br>Pulmonary parameters: yes
Oliveira et al., 2023, [51]	59<br>52.32 + 11.87<br>(34/25)<br>IG: 31<br>CG: 28	IG: mobility, stretching, breathing techniques, resistance, strength, balance and relaxation<br>CG: no training but received educational orientation and performed ADLs	12 weeks 24 sessions<br>2 sessions/week 60 min<br>warm up: 10 min (stretching, mobility, breathing techniques)<br>resistance: 20 min (walking)<br>strength: 15 min, 7 exercises 2 sets 10 rep., upper and lower limbs, progression in weights<br>balance: 5 min<br>relaxation: 10 min	Symptoms: n. m.<br>Quality of life: no<br>Physical fitness: no<br>Pulmonary parameters: n. m.
Ali et al., 2023, [52]	60<br>45.7±2.40<br>(31/29)<br>Group A: 30<br>Group B: 30	Group A: traditional physiotherapy program: aerobic exercise, muscle strengthening, and respiratory exercise<br>Group B: active cycle of breathing technique and traditional physiotherapy program	12 weeks<br>3 sessions/week<br>Breathing cycle: 2-3 times<br>Aerobic exercise: 20 min with daily increasing (HR, oxygen serration and Borg Scale measured in each session)<br>Resistance training: 10-45 min, 30-40% of 1RM to 80% of 1RM, 3 sets with 8-15 rep.<br>Diaphragmatic breathing exercise: 3 times/week 10 rep.<br>Pursed-lip abdominal breathing exercise: 3-4 times daily for more than 3 min	Symptoms: yes<br>Quality of life: n. m.<br>Physical fitness: yes<br>Pulmonary parameters: n. m.
Sanchez-Mila et al., 2023, [53]	200<br>(100/100)<br>IG: 100, 24±14, (51/49)<br>CG: 100, 40±22, (49/51)	IG: inspiratory muscle training with device and aerobic exercise<br>CG: traditional respiratory/diaphragmatic exercises and aerobic exercise	31 days<br>inspiratory muscle training: 5min/day, individual resistance levels based on initial assessment (MIP)<br>diaphragmatic exercise: sitting position<br>Aerobic exercise (walking): 40 min/session from day 2 to day 30, 1 session for 5 consecutive days, followed by a rest day, intensity of 60-75% HRmax and 50-60% VO_2max	Symptoms: yes<br>Quality of life: n. m.<br>Physical fitness: yes<br>Pulmonary parameters: yes
Szarvas et al., 2023, [54]	68<br>53.5<br>(29/39)	group exercise session: breathing techniques, chest mobility, muscle strengthening<br>low-intensity individual training: aerobic exercise breathing: respiratory muscle	14 days<br>group exercise: 2-3 sessions/day each 30 min<br>aerobic exercise: monitoring of HR and oxygen saturation<br>respiratory muscle strengthening: 2 times/day, 30 rep. 40% MIP	Symptoms: yes<br>Quality of life: yes<br>Physical fitness: yes<br>Pulmonary parameters: yes<br>No adverse events

		strengthening, stretching, active cycle breathing		
**PHYSICAL EXERCISE, BREATHING EXERCISE AND PACING**				
Jimeno-Almaz et al., 2023, [55]	80<br>45.3±8.0<br>(55/25)<br>concurrent training: 20<br>Inspiratory muscle training: 17<br>Combination: 23<br>CG: 20	concurrent training: resistance and endurance training<br>inspiratory muscle training: with threshold devise<br>combination: concurrent training and inspiratory muscle training<br>CG: self-management WHO	8 weeks<br>concurrent training: 3 sessions/week, in 2 sessions resistance: 3 sets 8 rep. with 4 exercises (50% 1RM) and moderate intensity variable training (4-6x 3-5 min at 70-80% HRR/2-3 min at 55-65% HRR and <16 RPE), 1 session light intensity continuous training 30-60 min, 65-70% HRR 11-12 RPE<br>Inspiratory muscle training: 2 standardized daily sessions 1 set 30 rep. ~60% MIP and 11-12 RPE<br>Combination: 3 sessions/week concurrent training and daily inspiratory muscle training<br>Control group: gradual increase of activity (Phase 1: RPE 0-1, Phase 2: RPE 2-3, Phase 3: RPE 4-5, Phase 4: RPE 5-7, Phase 5: RPE 8-10	Symptoms: yes<br>Quality of life: yes<br>Physical fitness: yes<br>Pulmonary parameters: n.m.<br>No adverse events
**PHYSICAL EXERCISE AND SOPHROLOGY**				
Vallier et al., 2023, [56]	17<br>54.8±16.0<br>(5/12)<br>Inpatient: 9, 53.1±13.9, (4/5)<br>Home-based: 8, 56.9±18.9, (1/7)	(inpatient- or home-based rehabilitation)<br>Both: walks, endurance sessions, gymnastics/muscular strength, sonography, medical consultation (dietician, psychologist, physician before and after rehabilitation)	4 weeks<br>walks: 4 times/week 60 min<br>endurance: 4 times/week 40 min, 10 min warm-up, 10 min cool-down, 20 min (at 90-100% HR achieved at the end of 6MWT)<br>gymnastic/muscular strength: 3 times/week<br>sophrology: 1 time/week<br>medical consultation: 1 time/week	Symptoms: yes<br>Quality of life: yes<br>Physical fitness: yes<br>Pulmonary parameters: yes
**PHYSICAL EXERCISE, BREATHING EXERCISE AND EDUCATIONAL SESSIONS**				
Ponce-Campos, et al., 2022, [57]	42<br>53.35<br>(17/25)	Physiotherapy protocol: breathing exercises, mobilization, relaxation, progressive strengthening exercise, teaching energy saving techniques, aerobic exercises, balance and coordination	4 weeks<br>12 sessions 3 sessions/week<br>Individualized according to the need of each patient<br>Aerobic exercises: week 2 55-60%, week 3 60-65%, week 4 70-75% of HRmax	Symptoms: yes<br>Quality of life: n. m.<br>Physical fitness: yes<br>Pulmonary parameters: yes
Rzepka-Cholasińska et al., 2024, [58]	90<br>61.65 ± 5.39<br>(49/41)	personalized rehabilitation program<br>low initial activity levels and low muscle strength (MRC < Grad3) or severe dyspnea (mMRC ≥ 3)<br>high-intensity interval training:<br>low to medium initial activity levels and medium muscle strength (MRX = Grad3) or with medium dyspnea (mMRC = 2):<br>higher initial activity levels and muscle strength (MRC > 4) or with mild dyspnea (mMRC = 1):<br>all groups: aerobic exercises, strength and resistance training, balance, breathing, respiratory exercises, stretching, education (5 min warm-up, 20 min proper part, 5 min calming phase)	6 weeks<br>3 sessions/week<br>30 min/session<br>Resistance exercise: week 1 and 2 1 session, week 3 and 4 2 sessions, week 5 and 6 3 sessions/week<br>Progression: each exercise 1 set 10 rep. to 3 sets 20 rep.<br>intensity: progression 30-60% HRR	Symptoms: yes<br>Quality of life: yes<br>Physical fitness: yes<br>Pulmonary parameters: n. m.

Mammi et al. 2023, [59]	50<br>53±11.4<br>(29/21)	outpatient rehabilitation program: manual physical therapy, soft tissue, kinesiotaping, stretching, flexibility and mobility, core stability, static and dynamic balance, endurance training, educational session	2 sessions/week<br>10 individual sessions for 45 min<br>10 rep.<br>Average: 14.3 ± 4.16 sessions<br>Endurance training with Borg Scale (for progression), every exercise with 10 rep.	Symptoms: yes<br>Quality of life: yes<br>Physical fitness: n. m.<br>Pulmonary parameters: n. m.
Pietranis et al., 2024, [60]	59<br>63.1±13.41<br>(37/22)	Rehabilitation program<br>Both groups: physiotherapeutic interventions: aerobic training, respiratory training, resistance training, overall fitness exercises, stretching, educational sessions<br>IG: resistance training using a respiratory muscle trainer<br>CG: placebo respiratory muscle trainer	6 weeks<br>Aerobic training: interval 4/2 min from 15-20 min to 30-45 min, 45-55% to 70-80% HRmax, progression in time and shortening rest phases<br>Respiratory training: 10-15 min<br>Resistance training: 10-15 min, major muscle groups, progression in rep. 8-10-12, individual intensity<br>Overall fitness and stretching: 10-15 min<br>Respiratory muscle trainer: 15-20 min, 6 sets of 6 maximally deep breaths	Symptoms: n. m.<br>Quality of life: n. m.<br>Physical fitness: n. m.<br>Pulmonary parameters: yes<br>No serious adverse events
Benzarti et al., 2022, [61]	14<br>61±4<br>Only male	Aerobic cycle endurance, strength training, balance, stretching, relaxation, breathing techniques and education	4 weeks<br>3 sessions/week,70 min<br>warm up 5 min<br>lower limbs strengthening: 45 min cycling (HR monitoring, target HR is HR in the end of 6MWT)<br>upper limb strengthening: 10 min with sets of 10 rep. with progression in load<br>balance: 5 min<br>relaxation: 5 min	Symptoms: yes<br>Quality of life: yes<br>Physical fitness: yes<br>Pulmonary parameters: n. m.
**PHYSICAL EXERCISE AND EDUCATIONAL SESSIONS**				
Daynes et al., 2021, [62]	30<br>58<br>(14/16)	Aerobic exercise (treadmill walking), strength training (upper and lower limbs), educational discussions	6 weeks<br>2 sessions/week<br>Borg breathlessness scale and rate of perceived exertion were used for progression	Symptoms: yes<br>Quality of life: yes<br>Physical fitness: yes<br>Pulmonary parameters: n. m.<br>No serious adverse events
Colas et al., 2023, [63]	38<br>46.9±12.7<br>(21/17)	aerobic exercise, resistance exercise, educational sessions	4 weeks<br>3 sessions/week each for 2h<br>2 sessions/week: aerobic exercise 90 min, resistance exercise 30 min<br>1 session: therapeutic education	Symptoms: n. m.<br>Quality of life: n. m.<br>Physical fitness: yes<br>Pulmonary parameters: yes
**MULTIDISCIPLINARY REHABILITATION PROGRAM**				
Halvorsen et al., 2024, [64]	20<br>62.35±14.02<br>(11/9)	HIT (50% treadmill walking, 25% overground walking, 25% stairs), pulmonary rehabilitation techniques (diaphragmatic breathing, inspiratory hold and stacked breathing, inspiratory muscle training with device), when indicated: occupational therapy, speech and language pathology	median 10 sessions in total<br>5 sessions/week<br>HIT: 4 sessions/week, 60 min/session, 70-85% of the age-predicted HRmax and RPE 14-17, monitoring of HR and oxygen saturation<br>breathing: 2 times/day, 5 sets of 6 breaths	Symptoms: n. m.<br>Quality of life: yes<br>Physical fitness: yes<br>Pulmonary parameters: yes<br>some adverse events
Gloeckl et al., 2021, [65]	50<br>(28/22)<br>Moderate: 24, 52	Endurance training, strength training, relaxation, ADL training, respiratory physiotherapy, occupational therapy,	3 weeks<br>Endurance: 5 days/week 10-20 min, 60-70% of peak work rate<br>strength training: 5 days/week 30 min, 3 sets 15-20 rep. with individual intensity	Symptoms: n. m.<br>Quality of life: yes<br>Physical fitness: yes

	Severe: 26, 66	psychological support, nutritional counselling, education	ADL training: 4-5 days/week 30 min<br>Nordic Walking or aqua fitness: 2 days/week 30 min<br>relaxation: 2 days/week 30 min<br>respiratory physiotherapy 2-4 days/week 30 min	Pulmonary Parameters: yes<br>No adverse events
Hasting et al., 2023, [66]	33<br>49.2±9.8<br>(24/9)	neuropsychological treatment, pacing, relaxation and mindfulness techniques, cognitive training, speech and language therapy, physiotherapy (strength, fitness training, mobilization, stretching, breathing exercises), psychoeducation, nutritional counseling, social and medical consultation	3 weeks<br>10 treatment days: 3-4 units/day<br>cognitive training: 2-3 sessions/week<br>medical consultation: once a week<br>Physiotherapy: daily, using pacing to adopt optimal activity rhythm (light to moderate intensity)	Symptoms: yes<br>Quality of life: yes<br>Physical fitness: n. m.<br>Pulmonary parameters: n. m.
Hayden et al., 2021, [67]	53<br>(28/25)<br>Group B: 54.0±9.9<br>(11/21)<br>Group C: 52.1±6.8<br>(17/4)	Physical training (endurance training, strength training, whole-body vibration training, inspiratory muscle training), respiratory physiotherapy, general physiotherapy, education session, medical diagnostics/supervision, psychological support, nutritional counseling, occupational therapy	3 weeks<br>Endurance training: 3-5 sessions/week for 30-60 min, individual duration and intensity based on initial 6MWT, controlled by RPE 4-6, oxygen saturation ≥90%, HR monitoring<br>Strength training: 2-3 sessions/week, 45-60 min, 3 sets with 10 rep., major muscle groups, individual duration and frequency<br>Whole-body vibration training: 7 sessions/week 3 sets 1-2 min/session intensity (16–26 Hz, 1.5–4 amplitude)<br>Inspiratory muscle training: 7 sessions/week, 21 min<br>respiratory physiotherapy: 2 sessions/week, 45 min<br>educational session: once 45 min	Symptoms: yes<br>Quality of life: yes<br>Physical fitness: yes<br>Pulmonary parameters: yes
Kesikburun et al., 2023, [68]	39<br>59.7±15.6<br>(15/24)	neuromuscular rehabilitation: mobilization, progressive muscle strengthening, balance and coordination, FES cycling<br>cardiopulmonary rehabilitation: aerobic exercise, muscle strengthening, breathing exercise<br>speech and language therapy, psychological support, occupational therapy, nutritional counseling	6 weeks 30 sessions<br>5 days/week, 1 hour/session<br>FES cycling: 2 sessions/week, 10 sessions, 30 min<br>aerobic exercise: 3-5 days/week for 4 weeks, 30 min<br>inspiratory muscle training: 2 times/day for 2 weeks, 20 min<br>monitoring of blood pressure, HR and oxygen saturation in each session	Symptoms: yes<br>Quality of life: yes<br>Physical fitness: n. m.<br>Pulmonary parameters: n. m.
Nopp et al., 2022, [69]	58<br>47<br>(25/33)	individualized endurance, strength, and inspiratory muscle training<br>education, psychosocial counseling, nutritional education, smoking cessation sessions	6 weeks<br>3 sessions/week, 3-4 hours each<br>60 rehabilitation sessions 50 min (56 therapeutic sessions [2 nutrition, 2 psychosocial counseling, 4 medical evaluations, rest physical exercise], 4 diagnostic and assessments)	Symptoms: yes<br>Quality of life: yes<br>Physical fitness: yes<br>Pulmonary parameters: yes<br>No adverse events
Grishechkina et al., 2023, [70]	113<br>58.4<br>(83/30)<br>IG: 25<br>CG 1: 25<br>CG 2: 29<br>CG 3: 34	IG: aquatic exercises, respiratory and motor exercises, social integration training, neuropsychologic sessions, LASER therapy, magnetotherapy<br>CG 1: eastern medicine techniques: climatotherapy, acupuncture, manual therapy, QuiGong breathing, exercise therapy, LASER therapy, magnetotherapy	IG: 7-8 sessions<br>CG 1: 7-8 sessions<br>CG 2: 10-15 sessions<br>CG 3: ??	CGs: more hospital admissions, need for specialist consultations and ambulance calls

		CG 2: respiratory and motor exercise therapy, physiotherapy combined with inhalation of mineral water, balneotherpy with dry carbon dioxide baths,pmagnetotherapy<br>CG 3: self-training and home-based physical exercise		
**MULTIDISCIPLINARY REHABILITATION PROGRAM (ROBOT)**				
Zasadzka et al., 2022, [71]	28<br>(10/18)<br>IG: 14, 69, (6/8)<br>CG: 14, 66, (4/10)	neuromuscular re-education techniques (PNF and Bobath therapy), coordination, balance, progressive endurance training, psychologist, speech and occupational therapy<br>IG: EMG-rehabilitation robot (isokinetic-isotonic training of selected muscle groups using reactive electromyography)	6 weeks<br>6 days/week IG: 75 min, CG: 120 min<br>Progressive endurance training: 30 min, 35-70% HRmax<br>PNF or Bobath therapy: 45 min<br>Occupational therapy: daily 30 min<br>Psychological and speech therapy: 5 sessions/week, 30 min<br>IG: EMG-rehabilitation robot (6 days/week, 45 min/day in 2 sessions)	Symptoms: yes<br>Quality of life: yes<br>Physical fitness: yes<br>Pulmonary parameters: n. m.<br>No adverse events with EMG-rehabilitation robot
Trzmiel et al., 2023, [72]	81<br>IG: 42, 67.38±8.46<br>CG: 39, 64.92±11.74	neuromuscular re-education techniques (PNF and Bobath therapy), coordination, balance, progres-sive endurance training, psychologist, speech and occupational therapy<br>IG: EMG-rehabilitation robot (isokinetic-isotonic training of selected muscle groups using reactive electromyography)	6 weeks<br>6 days/week IG: 75 min, CG: 120 min<br>Progressive endurance training: 30 min, 35-70% HRmax<br>PNF or Bobath therapy: 45 min<br>Occupational therapy: daily 30 min<br>Psychological and speech therapy: 5 sessions/week, 30 min<br>IG: EMG-rehabilitation robot (6 days/week, 45 min/day in 2 sessions)	Symptoms: n. m.<br>Quality of life: yes<br>Physical fitness: yes<br>Pulmonary parameters: n. m.
**TELEREHABILITATION**				
Calvo-Paniagua et al., 2022, [73]	68<br>48.5 ± 9.7<br>(42/26)	posture ergonomics, respiratory control, physical exercise (upper and lower limbs, core), aerobic exercise (walking), mobilization, motor control exercise, occupational therapy, diaphragmatic respiratory education	up to 7 weeks<br>3 sessions/week<br>40min<br>physical conditioning with increasing intensity	Symptoms: yes<br>Quality of life: yes<br>Physical fitness: yes<br>Pulmonary parameters: n. m.<br>No adverse events
Bileviciute-Ljungar et al., 2024, [74]	67<br>43<br>(52/15)	Breathing exercise, mindfulness, relaxation, muscle strength training (yoga and Qigong), exercise on one's own (videorecorded exercises or free-chosen physical activities), psychoeducation	8 weeks<br>3 days/week for 2 hours<br>3 hours exercise on one's own	Symptoms: yes<br>Quality of life: yes<br>Physical fitness: yes<br>Pulmonary parameters: n. m.<br>Some reported a worsening of symptoms
Estebanez-Pérez et al., 2022, [75]	32<br>45.93<br>(23/9)	Digital individual physiotherapy: patient education, aerobic exercise, strength and training exercises, breathing exercises, recommendations for secretion drainage and ventilatory techniques	4 weeks<br>Limit: 1 session/day<br>45-50 min<br>Walking, jogging or swimming: 3-5 sessions/week, 20-30 min low intensity, gradual increase of intensity and duration, depends on sensation of fatigue and/or dyspnea<br>Progressive Strength training: 3-5 sessions/week, 1-3 muscle groups 8-12 rep. increasing load (5-10%/week)	Symptoms: n. m.<br>Quality of life: n. m.<br>Physical fitness: yes<br>Pulmonary parameters: n. m.<br>No adverse events

Colas et al., 2022, [76]	15<br>52.1± 12.2<br>Both sexes<br>IG: 9<br>CG: 6	IG: personalized Telerehabilitation: aerobic exercise, resistance exercise, therapeutic education workshops, psychological and nutritional support<br>CG: traditional physiotherapy: delivery of a training booklet with tests results of the initial evaluation, psychological and nutritional support	4 weeks<br>IG: 1st week at hospital: 3 sessions 1 hour (45 min aerobic, 15 min resistance)<br>for 3 weeks at home: 3 sessions/week, 45 min aerobic (at VT1 progressed to intermittent work at VT2 [last week]), 15 min resistance (whole body circuit training, from light to moderate intensity) (progression: RPE 2-6), monitoring of HR and RPE (training stopped when HR >80% HRmax or RPE >6/10)<br>3 therapeutic educational workshops	Symptoms: yes<br>Quality of life: n. m.<br>Physical fitness: yes<br>Pulmonary parameters: yes<br>No adverse events
Reis et al., 2023, [77]	49<br>(26/23)<br>IG: 24, 50.2±13.7, (10/14)<br>CG: 25, 63.9±9.2, (16/9)	IG: ventilatory control training, aerobic exercise, muscle strengthening exercise, respiratory muscle training, flexibility, balance<br>CG: usual care: initial clinical evaluation, management of the therapeutic regimen, education, and training relative to their health status (only physical activity education)	12 weeks 3 sessions/week and<br>2 weeks One session/week (38 sessions of 60 min)<br>intensity was adapted according to the perception of dyspnea according to the modified Borg scale<br>aerobic exercise: 5-30 min RPE 4-5<br>strength training: 6-7 exercises, 2 sets 10 rep., RPE 5<br>inspiratory muscle training: 5 sessions/week, 2 blocks of 10 min<br>monitoring of HR and oxygen saturation	Symptoms: yes<br>Quality of life: yes<br>Physical fitness: yes<br>Pulmonary parameters: n. m.<br>No adverse events
Rodriguz-Blanco et al., 2023, [78]	48<br>(26/22)<br>IG: 24, 38.75±15.40, (13/11)<br>CG: 24, 42.58±11.40, (13/11)	IG: therapeutic exercise program<br>CG: relative home rest, consisting of ADLs	14 days<br>1 session/day<br>IG: 10 breathing and strength-based exercises<br>12 rep. per exercise for 30 min/day<br>Intensity: rep. modified (Borg scale)	Symptoms: yes<br>Quality of life: n. m.<br>Physical fitness: yes<br>Pulmonary parameters: n. m.
McGregor et al., 2024, [79]	585<br>56±12<br>(305/280)<br>IG: 298<br>CG: 287	IG: online 1:1 consultation, supervised live online group exercise, live online group psychological support, on-demand library of physical activity (circuit, Interval, aerobic, breathing exercise, Pilates, yoga)<br>CG: usual care: best practice usual care consisting of online, one-to-one consultation with a trained practitioner (information and advices)	1-8 weeks<br>IG: 1 one-to-one consultation, 30-60 min<br>1 weekly live online group exercise sessions: 45-60 min<br>1 weekly live online group psychological support sessions: 60 min<br>on demand library of physical activity: 1-2 session/week, 30 min, moderate to high intensity exercises<br>CG: one session 30 min	Symptoms: yes<br>Quality of life: yes<br>Physical fitness: n. m.<br>Pulmonary parameters: n. m.<br>Several and serious adverse events
**VR TECHNOLOGY**				
Rutkowski et al., 2022, [80]	32<br>57.8±4.9<br>(20/12)	Both groups: exercise capacity training (cycling), walking training, resistance training, general fitness exercise, circuit training, breathing exercise, relaxation, techniques for removing secretions from the bronchial tree, inhalations<br>VR group: head-mounted display employed during cycling and relaxation	3 weeks<br>5 sessions/week<br>cycling: 30 min, intensity: model A: 80% of the submaximal HR, B: 70%, C: 60%, D: 20–30% (based on patient's submaximal exercise tolerance test results)	Symptoms: yes<br>Quality of life: no<br>Physical fitness: yes<br>Pulmonary parameters: n. m.
Rutkowski et al., 2023, [81]	32<br>57.8±4.92<br>(20/12)	Both groups: exercise capacity training (cycling), walking training, resistance training, general fitness exercise, circuit training, breathing exercise, relaxation, techniques for removing secretions from the bronchial tree, inhalations	3 weeks<br>5 sessions/week<br>cycling: 30 min, intensity: model A: −90% of HRpeak 6-minute walk HR, model B: −80%, model C: −70%, and model D: 20–30% and progression: Model A: 50W-150W, Model B: 50W- 100W, Model C: 30W- 80W, Model D: −30W (based on patient's distance and dyspnea rating in 6MWT)	Symptoms: yes<br>Quality of life: n. m.<br>Physical fitness: yes<br>Pulmonary parameters: no

		VR group: head-mounted display employed during cycling and relaxation		
**PACING**				
Parker et al., 2023, [82]	31<br>47±9<br>(22/9)	WHO Borg CR-10 pacing protocol for physical activity guidance	6 weeks<br>Using WHO Borg CR-10 pacing protocol, individuals had to stay at each phase for a minimum of 7 days and can use example activities<br>Phase 1: "Preparation for return to exercise" (RPE 0-1)<br>Phase 2: "Low-intensity activity" (RPE 2-3)<br>Phase 3: "Moderate-intensity activity" (RPE 4-5)<br>Phase 4: "High-intensity exercises" (RPE 5-7)<br>Phase 5: "Return to baseline" (RPE 8-10)	Symptoms: n. m.<br>Quality of life: yes<br>Physical fitness: n. m.<br>Pulmonary parameters: n. m.<br>less average number of PESE episodes

* f, female; m, male; n. m. not measured; rep., repetition; HRR, heart rate reserve, HRmax, maximal heart rate; HRpeak, peak heart rate; IG, intervention group; CG, control group; MIP, maximal inspiratory pressure; ADLs, activities of daily living; 6MWT, 6-minute walk test; CR-10 Borg scale, Category-Ratio-10 Borg scale; RPE, Borg Rating of Perceived Exertion scale; 1RM, one repetition maximum; HIIT, high intensity interval training; HIT, high intensity training; PNF, proprioceptive neuromuscular facilitation; PESE, Post-Exertional Symptom Exacerbation; FES cycling, functional electrical stimulation cycling; VT1, ventilatory threshold 1; VT2, ventilatory threshold 2
